# Machine learning-based predictions and analyses of the creep rupture life of the Ni-based single crystal superalloy

**DOI:** 10.1038/s41598-024-71431-1

**Published:** 2024-09-05

**Authors:** Fan Zou, Pengjie Liu, Yanzhan Chen, Yaohua Zhao

**Affiliations:** 1 School of Traffic & Transportation Engineering, Central South University, Changsha, 410083 China; 2 School of Intelligent Manufacturing and Mechanical Engineering, Hunan Institute of Technology, Hengyang, 421002 China

**Keywords:** Creep property prediction, XGBoost, Shapley additive explanations, Sparrow optimization algorithm, Computational methods, Mechanical engineering

## Abstract

The evaluation of creep rupture life is complex due to its variable formation mechanism. In this paper, machine learning algorithms are applied to explore the creep rupture life span as a function of 27 physical properties to address this issue. By training several classical machine learning models and comparing their prediction performance, XGBoost is finally selected as the predictive model for creep rupture life. Moreover, we introduce an interpretable method, Shapley additive explanations (SHAP), to explain the creep rupture life predicted by the XGBoost model. The SHAP values are then calculated, and the feature importance of the creep rupture life yielded by the XGBoost model is discussed. Finally, the creep fracture life is optimized by using the chaotic sparrow optimization algorithm. We then show that our proposed method can accurately predict and optimize creep properties in a cheaper and faster way than other approaches in the experiments. The proposed method can also be used to optimize the material design across various engineering domains.

## Introduction

The nickel-based single-crystal superalloy is the primary material that is used in aero-engine and gas turbine blades with excellent high-temperature performance^1^. To meet the design requirements of high-performance aero-engines, research and development activities with respect to nickel-based single-crystal superalloys are of great significance. In the current study, the creep property of the given material greatly limits the life cycle of the product. This is because the creep deformation and fracture of the material under high-temperature stress cause the product to fail^2^. Therefore, creep life is defined as the core research object of this paper because of its high scientific value. Even though the creep rupture life can be determined experimentally, the required measurements are often time-consuming and laborious due to lengthy creep tests and the high cost of alloy manufacturing, especially in cases when the alloy under study consists of noble metal elements such as Ru, Ti, and Ta^3^.

The creep property of an alloy is related to many variables that can be grouped into four categories: chemical composition factors, processing parameters, test conditions, and microstructural factors. The main chemical compositions of nickel-based superalloys are highly varied^4^, including nickel (Ni), rhenium (Re), cobalt (Co), chromium (Cr), and other alloy components. Additionally, the processing parameters, including the solution treatment time, heating temperature, test stress, and other parameters, are extremely complicated. The microstructural factors can be calculated from the chemical composition factors and processing parameters.

Since the experimental method is unable to meet the requirements of alloy design, several theoretical methods for accelerating the prediction of the creep fracture life spans of alloys have been proposed. Dang et al.^5^ performed creep fracture tests on three Mg–Al–Ca diecast alloys and used the Larson–Miller parameter (LMP) to predict the creep life. The results showed that the method performed well for specific temperature and stress levels. Li et al.^6^ investigated the creep properties of the K417 alloy at different high-temperature conditions and developed a physical analysis model to describe the influence of microstructural factors. As an example of using the time–temperature parameter (TTP) method, Larson and Miller^7^ developed a predictive model for creep life at low temperatures. Similarly, an isothermal method^8^ was proposed to describe the relationship between the creep property and stress under the conditions of constant temperature and changing stress for inferring the creep life of an alloy indirectly. In conclusion, the above traditional theoretical methods can achieve rapid creep life predictions, but they cannot reveal the complicated functional relationships between the creep life and its many influencing factors. Therefore, the accuracy of such models is limited.

With the development of big data and artificial intelligence, machine learning has been successfully applied to material performance prediction^9–11^, the discovery of new materials^12^, and other applications^13–16^. Data-driven approaches tend to be superior to other methods in terms of time efficiency and predictive performance. Since machine learning can learn the complex functional relationships between material properties and many factors, it is suitable for the task of predicting creep rupture life. Venkatesh and Rach^17^ developed and demonstrated a new method for life prediction using a BP (backward propagation) neural network. The results showed that the prediction accuracy of this method was significantly improved over that of linear models. By adopting the Bayesian neural network and Markov-chain Monte Carlo method, Yoo et al.^18^ predicted creep fracture life with an accuracy of 93.2% and ranked the importance of the constituent elements of the tested alloy.

**Fig. 1:**
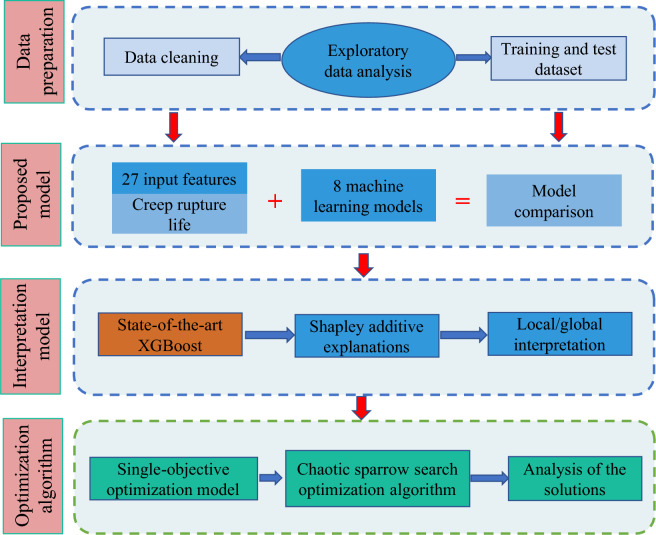
Overall framework of this study.

This paper not only predicts creep life but also explains the results of the predicted creep life and finally optimizes the chemical compositions and processing parameters by an optimization algorithm. The remainder of the paper is structured as follows. In “Machine learning methods” section, we use machine learning algorithms to build a mapping function between creep rupture life and 27 features, including chemical composition factors, processing parameters and microstructural factors. In “Explanatory methods for interpreting creep rupture life” section, a novel method, Shapley additive explanations (SHAP), is introduced to interpret the predictive results regarding creep rupture life. In “Optimization based on improved sparrow search algorithm” section, the chaotic sparrow optimization algorithm is applied to improve the creep rupture life and achieve the best solution. Finally, conclusions are provided in “Conclusions” section. The overall framework of this paper is shown in Fig. 1.

## Machine learning methods

### Acquisition of the data structure

**Fig. 2:**
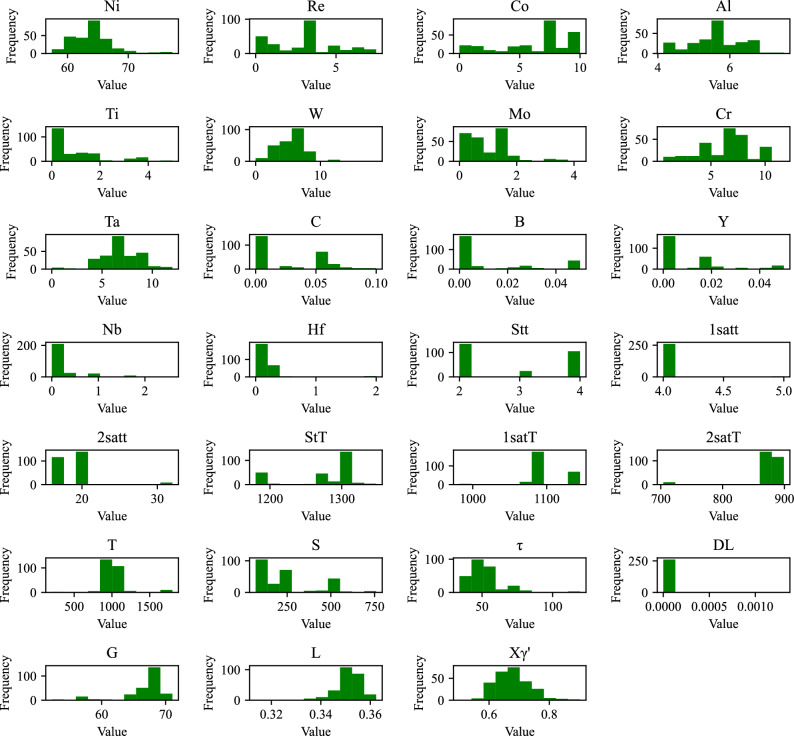
The histogram distribution for the 27 input features.

The creep rupture life dataset used in this study is obtained from previous publications^19–21^ and consists of 264 instances of superalloys. Each sample in the dataset contains 27 features describing its physical properties, which can be classified into the following four categories: chemical composition factors, processing parameters, test conditions and microstructural factors. A detailed description of the 27 features in the creep dataset is shown in Table 1. The distribution histograms of the 27 input features, as depicted in Fig. 2, reveal that certain features such as (1satt, 2satt, and 2satT) exhibit a high degree of variability and pronounced heterogeneity. Such characteristics can introduce several challenges to the predictive tasks in machine learning. The inherent complexity of these features may necessitate the employment of more sophisticated modeling techniques that can accommodate the diverse data patterns. Additionally, the variability could lead to increased model sensitivity to feature scaling and parameter tuning, requiring meticulous calibration to achieve optimal performance.

**Table 1:** Description of the data features.

Index	Symbol	Description	Minimum content	Maximum content
1	Ni	Nickel	57.4135	77.26
2	Re	Rhenium	0	7.5
3	Co	Cobalt	0	10
4	Al	Aluminum	4.15	7.5
5	Ti	Titanium	0	5
6	W	Masurium	0	18.6
7	Mo	Molybdenum	0	4.21
8	Cr	Chromium	0.6	11.73
9	Ta	Tantalum	0	12
10	C	Carbon	0	0.1
11	B	Boron	0	0.05
12	Y	Yttrium	0	0.05
13	Nb	Peloponium	0	2.59
14	Hf	Hafnium	0	2
15	Stt	Solution treatment time (h)	2	4
16	1satt	The first-stage aging treatment time (h)	4	5
17	2satt	The second-stage aging treatment time (h)	16	32
18	StT	Solution treatment temperature (°C)	1180	1348
19	1satT	The first-stage aging treatment temperature (°C)	982	1145
20	2satT	The second-stage aging treatment temperature (°C)	704	899
21	T	Test temperature (°C)	204	1800
22	*S*	Test stress (MPa)	70	759
23	$\Gamma$	Stacking fault energy (${\text{mJ}}/{\text{m}}^{2}$)	34.00666	119.4323
24	${D}_{L}$	Diffusion coefficient (${\text{m}}^{2}/{\text{s}}$)	1.41E−25	0.00131
25	$G$	Shear modulus (GPa)	52.20007	71.05828
26	$L$	Lattice parameter (nm)	0.313652	0.362381
27	${\text{X}}^{{\gamma \prime}}$	Mole fraction of the ${\gamma }{\prime}$ phase	0.50187	0.90197

### Data standardization

Data standardization is often the first step in machine learning tasks. As shown in Table 1, a total of 27 features that affect creep life vary widely in terms of their value ranges. For example, the value of the diffusion coefficient ${D}_{L}$ ranges from 1.41E−25 to 0.00131 ${\text{m}}^{2}/{\text{s}}$, while the solution treatment temperature ranges from 1180 to 1348 °C. Therefore, this may become an obstacle during the model training process. Here, we use the min–max scaling method^22^ to achieve isometric scaling of the original data. The min–max scaling method is employed to preprocess the 27 features, hence, all descriptors are mapped to [0, 1]. The method is defined as follows:

1 $${X}^{*}=\frac{X-{X}_{min}}{{X}_{max}-{X}_{min}},$$

where ${X}_{min}$ and ${X}_{max}$ represent the lower and upper limits of a feature $X$, respectively. Furthermore, from the original dataset, it can be seen that the creep life spawns of the samples are distributed in the interval from 30 to 5180 h. We use logarithmic scaling for the creep life, as shown in Eq. (2). This enables us to efficiently convert the predicted creep life. Finally, the value of the feature after data normalization is used as an input parameter for the machine learning model mentioned in Table 2.

2 $${Y}^{*}=\text{log}\left(Y\right).$$

### XGBoost algorithm

**Fig. 3:**
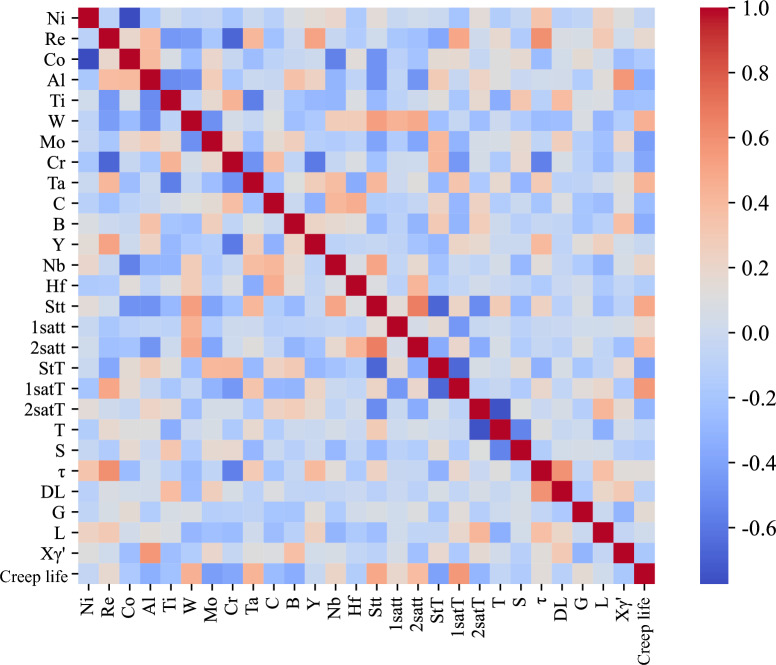
Heat map based on the Pearson correlation coefficient.

Before the machine learning task is completed, we perform a Pearson correlation analysis of the creep dataset. Figure 3 shows a heat map that is an indication of the relationships between the 27 features and the creep rupture life. This is, however, only a simple linear analysis and may not be able to explore the more complex nonlinear relationships between the variables. Therefore, several machine learning models with strong representational capabilities are discussed in the following sections.

**Fig. 4:**
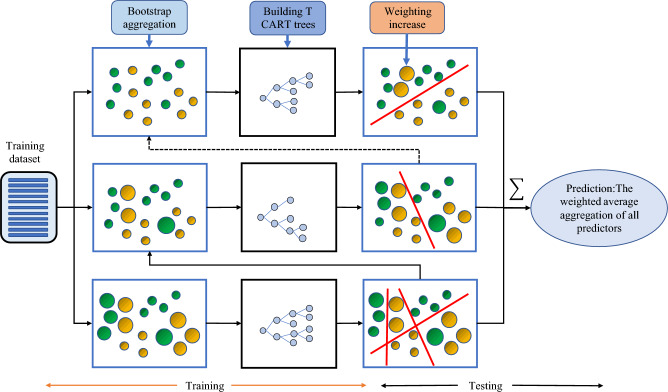
The schematic diagram of XGBoost.

XGBoost (eXtreme Gradient Boosting) is a successful machine learning library based on the gradient boosting algorithm proposed by Chen^23^. It has been extensively used in industry due to the portability of the distributed version and its powerful predictive capabilities. As an improved GBDT (Gradient Boosting Decision Tree) algorithm^24^, XGBoost consists of a large number of decision trees strung together to handle classification and regression tasks accurately and efficiently. Compared with GBDT, XGBoost is mainly optimized in the following aspects. First, XGBoost adds a second-order Taylor expansion to the cost function, which improves the accuracy of the algorithm. Second, the regular penalty term^25^ is utilized in the cost function to reduce the model complexity and prevent overfitting. The Fig. 4 visually illustrates the procedural workflow of the XGBoost algorithm, beginning with the initial step of “Bootstrap aggregation”. This phase involves the utilization of resampling techniques to generate multiple subsets from the original dataset, encompassing a diverse range of classes. These subsets serve as the foundation for the subsequent stage known as ‘CART Trees’, which includes classification and regression trees, are binary models that are constructed and intertwined with each of the data subsets. The subsequent phase, termed ‘Weighting increase’, embodies the iterative nature of gradient boosting. This process dynamically adjusts the decision boundaries by focusing on and augmenting the weights assigned to misclassified points from previous iterations, thereby enhancing the overall accuracy of the model. The final stage cleverly combines the fitting results obtained from all the CART Trees generated throughout the workflow. By employing a weighted average of these results, the XGBoost model is capable of formulating an optimized and robust prediction.

### Empirical analysis and comparisons with other models

In this study, the dataset is split into training and test sets using a 10-fold cross-validation method. This method randomly divides the dataset into 10 subsets. Each time one of these subsets is considered as the test set, the remaining 9 subsets are used as the training sets. Using this approach, the machine learning model can be trained and tested 10 times to obtain a more comprehensive evaluation index. To reflect the accuracy of the predictive models, three evaluation parameters are adopted in this paper^26^, including the coefficient of determination (${R}^{2}$), mean absolute error ($\text{MAE}$), and root mean squared error ($\text{RMSE}$). Suppose that ${y}_{1}$,${y}_{2}$,$\cdots {y}_{n}$ are the actual values, ${\widetilde{y}}_{1}$,${\widetilde{y}}_{2}$,$\dots {\widetilde{y}}_{n}$ are the predicted values, and $\overline{y }$ is the mean of ${y}_{i}$; then,

3 $${R}^{2}=1-\frac{\sum_{i=1}^{n} {\left({\widetilde{y}}_{i}-{y}_{i}\right)}^{2}}{\sum_{i=1}^{n} {\left(\overline{y }-{y}_{i}\right)}^{2}},$$

4 $$\text{MAE}=\frac{1}{n}\sum_{i=1}^{n} \left|{\widetilde{y}}_{i}-{y}_{i}\right|,$$

5 $$\text{RMSE}=\sqrt{\frac{1}{n}\sum_{i=1}^{n}{\left({\widetilde{y}}_{i}-{y}_{i}\right)}^{2}.}$$

Without the loss of generality, we establish other classic predictive models for a performance comparison, including a decision tree^27^, a support vector machine^28^, a neural network^29^, a random forest^30^ and LightGBM^31^ and so on. Table 2 provides a brief description of each model.

**Table 2:** Introduction to different machine learning models.

Models	Notes
LR	Linear regressor
KNN	K nearest neighbor
SVM	Support vector machine
ETR	Extra tree regressor
DT	Decision tree
GB	Gradient boosting regressor
NN	Neural network
RF	Random Forest
LightGBM	Light gradient boosting machine
XGBoost	eXtreme gradient boosting

Here, we implement the methods in Table 2 using the Scikit-learn library^32^ with version 3.7 of Python, which is available at https://www.python.org/. In addition, XGBoost and LightGBM are implemented in Python using the XGBoost and LightGBM libraries, respectively. To ensure optimal performance of these models, we utilized the Optuna library^33^ for hyperparameter tuning. Optuna is a state-of-the-art framework for automated hyperparameter optimization, which allowed us to systematically search for the best hyperparameters for each model. The test platform includes a device equipped with an AMD Ryzen-7 4800H CPU and 16 GB of RAM. The above 10 machine learning models are trained and tested separately by employing 10-fold cross-validation.

**Fig. 5:**
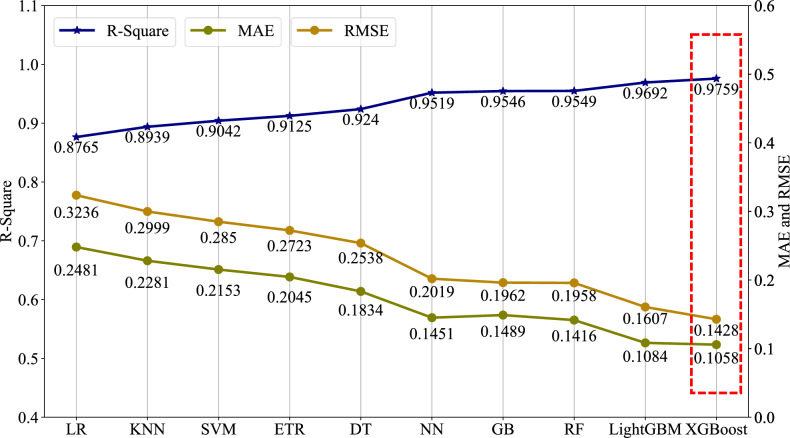
Results of the creep rupture life prediction models, including their ${R}^{2}$, $RMSE$ and $MAE$ values. A model with a larger ${R}^{2}$ value and lower of $RMSE$ and $MAE$ values has higher fitting accuracy.

The line chart in Fig. 5 shows the ${R}^{2}$, $\text{RMSE}$ and $\text{MAE}$ distributions corresponding to the 10 machine learning models used in this paper. Figure 5 shows that complex machine learning models (i.e., NN, GB, RF, LightGBM and XGBoost) achieve high prediction accuracy, and their corresponding ${R}^{2}$ values are larger than 0.95. The results also show that the prediction performance of LightGBM and XGBoost is better than that of the other models. In particular, for XGBoost, ${R}^{2}=0.9759$, $\text{RMSE}=0.1428$, and $\text{MAE}=0.1058$; these results are better than those of the other models. It is also seen that the performance of the three relatively simple machine learning models (LR, KNN, and SVM) is poor, and their ${R}^{2}$ values are low. Due to the obvious nonlinear relationships between the creep rupture life and the 27 features examined in this study, these three simple models are simply unable to reveal the complex mapping function relationships.

**Fig. 6:**
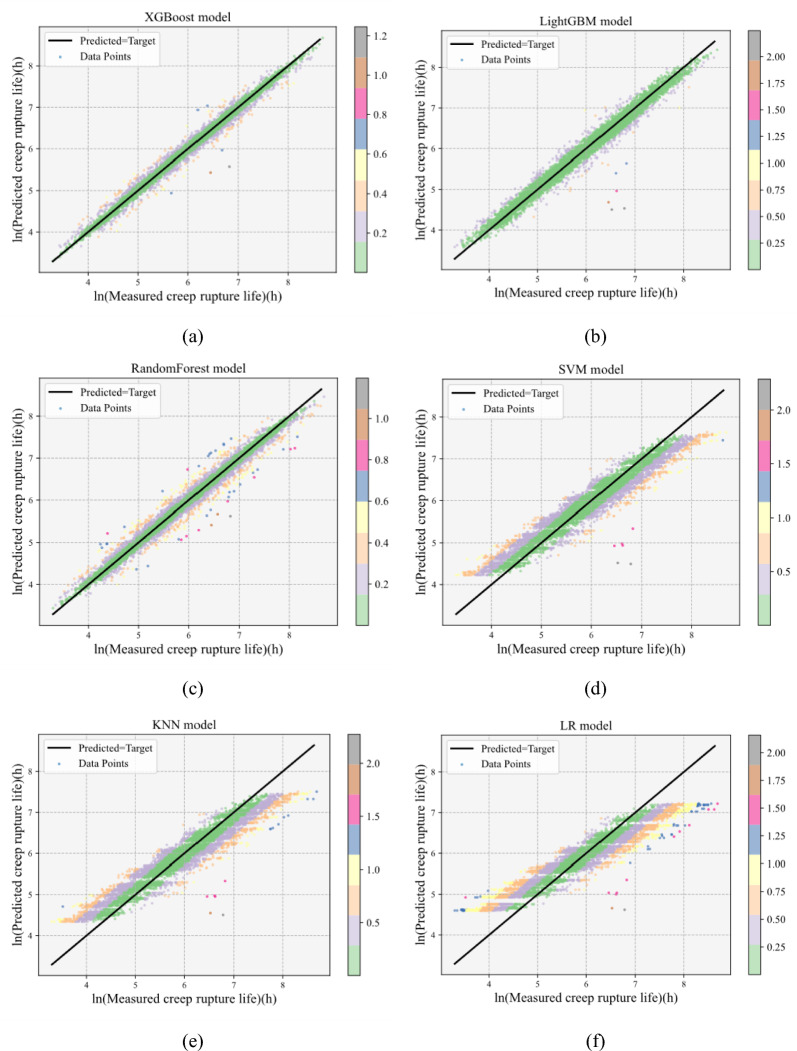
Predictive performance of the six machine learning models on the creep dataset. *Note*: The X-axis and Y-axis represent the actual and predicted values, respectively. The color bar represents the absolute error between predicted and actual values.

Finally, we utilize scatter plots to illustrate the fitting performance of the top three best-performing models and the bottom three underperforming models. As shown in Fig. 6, the experimental value of the creep rupture life is represented on the X-axis, and the predictions of each model are shown on the Y-axis. Each graph contains a 1:1 straight line representing a perfect fit. The closer the scatter points to the straight line, the better the fit of the model. It can be seen that the prediction effect of the XGBoost model is the best. Therefore, in this paper, we use XGBoost as the final model for the prediction of the creep life.

## Explanatory methods for interpreting creep rupture life

In machine learning, accuracy, complexity, and interpretability are the three most important concepts. In general, complexity and interpretability are in opposition to each other^34^. The XGBoost model used in this research has strong fitting capabilities due to its complex structure. However, due to its large number of parameters and complex working mechanisms, XGBoost is often regarded as a black-box model. To reveal the internal mechanisms of the 27 features that affect the creep rupture life, this section attempts to explain the XGBoost model using model interpretation methods.

### Principles of the SHAP method

Model interpretability is divided into two categories: global interpretability and local interpretability. Global interpretability is based on the entire studied dataset to help people understand the internal working mechanism and complex logic of the model, while local interpretability analyzes the specific decision-making process of the model for a single sample. In this paper, SHAP is used to improve the interpretability of the machine learning model. SHAP is a method proposed by Lundberg et al.^35^ that provides global and local interpretations and further possesses a solid theoretical foundation relative to those of other model interpretation methods. SHAP assigns an importance value to each feature of the test learning model. This facilitates the provision of a reliable explanation for a complex predictive model. The key idea of SHAP is to use a surrogate model $g$ to represent the black-box model $f$ that is hard to directly explain. The explanatory model $g$ is defined as a simple linear function, as expressed in Eq. (6):

6 $$f\left(x\right)\approx g\left({z}^{\prime}\right)={\phi }_{0}+\sum_{i=1}^{M}{\phi }_{i}{z}_{i}^{\prime},$$

where $f(x)$ is the original complex machine learning model’s prediction function, which takes an input $x$ and outputs a prediction, $g({z}^{\prime})$ is an approximation of the function $f(x)$, which is constructed to be more interpretable. It is a linear function of the features ${z}^{\prime}$, which are some transformed or perturbed versions of the original features $x$. ${z}_{i}^{\prime}\in {\left\{\text{0,1}\right\}}^{M}$ denotes a feature being observed (${z}_{i}^{\prime}=1$) and the unknown feature (${z}_{i}^{\prime}=0$); $M$ represents the total number of features, which is equal to 27 in this paper; ${\phi }_{i}$ represents the contribution value of the $i$-th feature to the model prediction result, and the sum of each feature’s contribution is similarly equal to the output $f(x)$ of the original model.

In SHAP, the Shapley value explanation is represented as an additive feature attribution method, i.e., a linear model. This view also connects the LIME (Local Interpretable Model-Agnostic Explanations)^36^ and Shapley values^37^. In previous studies, it was shown that Shapley values represent a unique solution that can satisfy three properties at the same time: symmetry, the dummy property, and additivity. To calculate the Shapley value of each feature, the mathematical expectation of the function conditioned on a subset of the input features is defined as $E[f(x){|x}_{S}]$. The contribution value of the i-th feature (${\phi }_{i}$) is a weighting of all mathematical expectations, as shown in Eq. (7):

7 $${\phi }_{i}=\sum_{S\subseteq N\backslash \{i\}}\frac{\left|S\right|!(\left|N\right|-\left|S\right|-1)!}{\left|N\right|!}[{f}_{x}(S\bigcup \{i\})-{f}_{x}(S)],$$

where ${\phi }_{i}$ represents the average contribution of feature $i$ to the prediction function, the summation is taken over all subsets $S$ of the set $N$ excluding feature $i$. The term $\left|S\right|$ denotes the cardinality of subset $S$, and $\left|N\right|$ is the total number of features. The function ${f}_{x}(S)$ is the prediction value of the model when only the features in subset $S$ are considered. The coefficient $\frac{\left|S\right|!(\left|N\right|-\left|S\right|-1)!}{\left|N\right|!}$ is a weight that reflects the proportion of subsets that include $S$ in all possible subsets of $N$, ensuring that each feature's contribution is fairly distributed.

### Local interpretation based on individual samples

The most prominent advantage of SHAP is that it can correspond to the influence of the features in each sample, and it can also show its positive and negative effects of these features. First, we show how SHAP values are locally attributed to individual samples in the creep dataset.

**Fig. 7:**
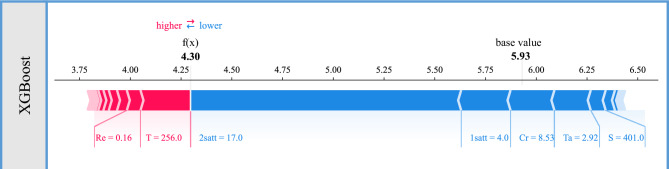
Explanation for the first sample obtained using the SHAP method.

We take the first sample in the dataset as an example to explore the impacts of these features on the model output. Figure 7 shows how each feature contributes to the predictive result by pushing it from the base value, which is defined as the average value of all sample predicted values in the dataset. In this example, this base value is equal to 5.93, while the predictive result of the XGBoost model is equal to 4.30. As shown in Fig. 7, the red and blue arrows indicate the positive and negative SHAP values of the features, respectively. On the right side of the output value (f(x)), the feature represented in blue in the graph pushes the predicted value toward lower values, while on the left side of the output value, the feature represented in red pushes the predicted value toward larger values. The longer the length of the arrow in the figure, the greater the contribution of the corresponding feature is. We see that 2satt (17.0) and 1satt (4.0) are related to the negative SHAP values, and a consequent reduction in the creep rupture life is expected. On the other hand, Re (0.16) and especially T (256.0) are related to the positive SHAP values, and the creep rupture life is expected to increase.

### Global interpretation based on all the samples

**Fig. 8:**
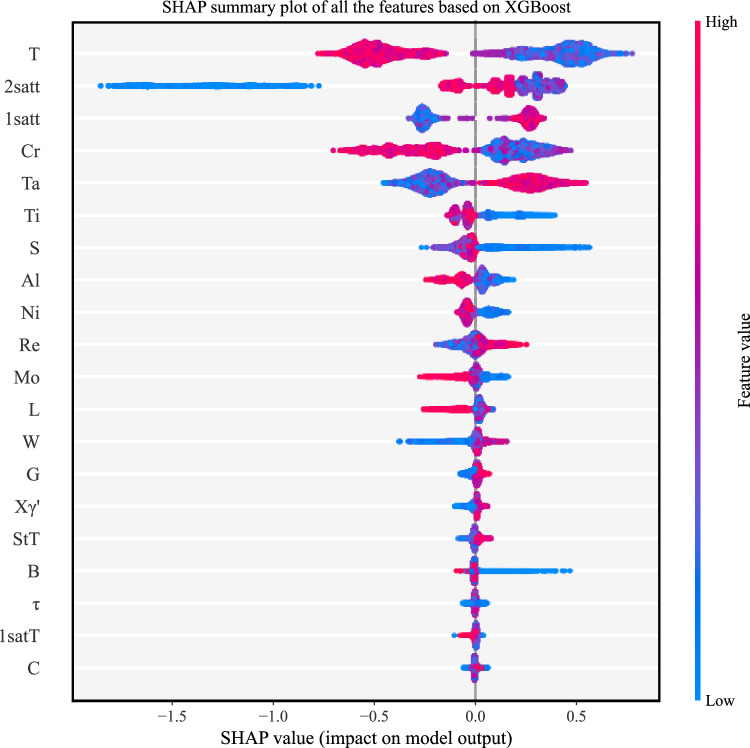
SHAP summary plot of the XGBoost model.

**Fig. 9:**
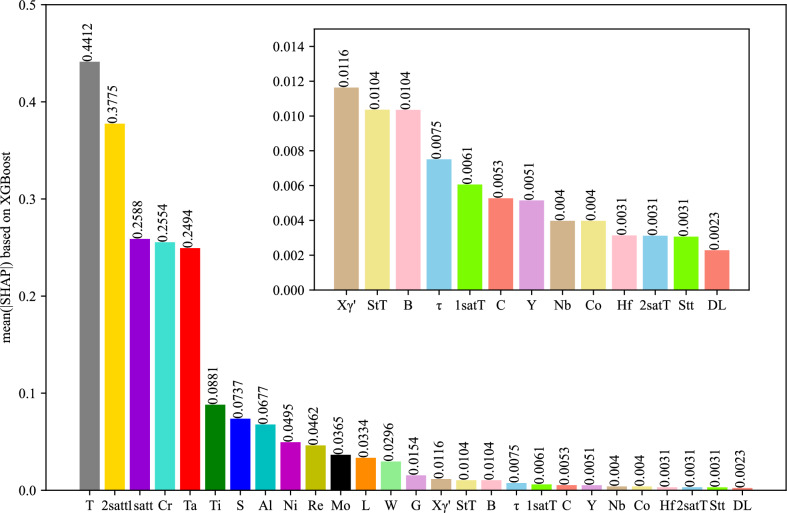
Feature importance rankings for predicting the creep rupture life.

The global interpretation is the aggregation of the local interpretation information of all samples so that it can fully reflect how each feature affects the creep life. For each sample, a set of SHAP values can be generated, and Fig. 8 shows the distribution of SHAP values for each feature based on the entire dataset. The Y-axis in the graph represents all features are ranked by their global contributions $\sum\nolimits^{N}_{j=1}\left|{{\phi }_{i}}^{(j)}\right|$, and the X-axis represents the SHAP values ${{\phi }_{i}}^{(j)}$. Each point represents a sample, from low (blue) to high (red), with different colors representing the values of the features. In addition, the importance of each feature is ranked from top to bottom. In Fig. 8, it can be seen that XGBoost generates the following four important factors for creep rupture life prediction: the test temperature (T); second-stage aging treatment time (2satt); first-stage aging treatment time (1satt); and mass percent of Cr (Cr). Furthermore, the test temperature is an extremely important feature that is negatively correlated with the creep rupture life. For a high test temperature (such dots in the graph are shown in red), the SHAP values are negative, and for a low test temperature (such dots in the graph are shown in blue), the SHAP values are positive. Shortening the experimental time can help to increase the creep rupture life. According to the same analysis, the contribution of each feature to the creep life can be discovered. It is worth mentioning that although the second-stage aging treatment time is negatively correlated with the creep rupture life, it is seen in Fig. 8 that a short second-stage aging time results in a reduction in the creep life. In terms of chemical composition, Cr has the most obvious effects on the creep rupture life. Figure 8 shows that the creep fracture life is negatively correlated with Cr content because an increase in Cr content compromises the phase stability of the alloy and thus affects the creep resistance of the alloy. In addition, we can also find that increasing Ta, Re, and W, three refractory elements, results in increasing the creep rupture life. This is because these elements can increase the dissolution temperature of the ${\gamma }^{\prime}$ phase and improve the temperature bearing capacity. To quantitatively analyze the contribution of each feature, we obtain the average of the absolute values of the SHAP values for each feature, and the feature importance rankings are shown in Fig. 9. These feature importance rankings not only enhance the transparency and interpretability of the black-box machine learning models but also guide the design of further experiments for optimizing creep rupture life.

## Optimization based on improved sparrow search algorithm

In this section, the XGBoost model is used as the mapping function between the creep life and the main variables, which is also the objective function. We then establish a single-objective optimization model that maximizes the creep rupture life. To enhance the global search ability and convergence speed of the model, a chaotic sparrow search optimization algorithm is proposed by importing the Tent chaotic sequence and Gaussian mutation.

### Model establishment

Here, we choose all 27 features as decision variables and XGBoost as the mapping function. Let $X=[{X}_{1},{X}_{2},{X}_{3},\cdots {X}_{n}](n=27)$ denote 27 features which can be classified into four categories, that is, chemical composition factors, processing parameters, test conditions and microstructural factors. Furthermore, $f\left(X\right)$ demonstrates the creep rupture life predicted by the XGBoost model. As a result, the optimization algorithm can be constructed as the following mathematical model.

$$\text{max}\left(f\left(X\right)\right)$$

8 $$s.t\left\{\begin{array}{l}{{X}_{1min}\le X}_{1}\le {X}_{1max},\\ {{X}_{2min}\le X}_{2}\le {X}_{2max},\\ {{X}_{3min}\le X}_{3}\le {X}_{3max},\\ \vdots \\ {{X}_{nmin}\le X}_{n}\le {X}_{nmax} \left(n=27\right),\\ \sum_{i=1}^{14}{X}_{i}=100.\end{array}\right.$$

where ${X}_{nmin}$ and ${X}_{nmax}$ are the lower and upper bounds according to the raw dataset, respectively, and their specific values are shown in Table 1. Notably, an equality constraint is established to ensure that the total sum of the 14 chemical element contents equals 100%. To solve this model, we employ an improved heuristic optimization algorithm which will described in the next section.

### Solution of the model

How to find an optimal set of parameter combinations in a high-dimensional search space is the problem to be solved by combinatorial optimization, and this section uses an optimization algorithm to determine the optimal combination of chemical composition factors and processing parameters. Currently, heuristic optimization algorithms are widely applied in the engineering field because of their simplicity and practicality. The main idea of such algorithms is to search for the optimal solution within a certain range by simulating the collective behavior of decentralized systems, e.g., in nature. Many swarm intelligence optimization algorithms have been proposed based on the swarm behaviors of ants, birds, bees, wolves, whales, sparrows, and other intelligent creatures. The sparrow search algorithm (SSA) proposed by Xue et al.^38^ in 2020 is a new type of swarm intelligence optimization algorithm. Compared with other algorithms, such as the particle swarm optimization (PSO)^39^, ant colony optimization (ACO)^40^, and gray wolf optimization (GWO) algorithms^41^, it provides higher search accuracy, faster convergence, and higher levels of stability and robustness. Therefore, here, we use an improved SSA algorithm to improve the creep rupture life.

#### Standard SSA algorithm

The sparrow search algorithm (SSA) is inspired by the foraging and antipredation behaviors of sparrows. Its core idea is to abstract the foraging process of sparrows as a producer-scrounger model with reconnaissance and early warning mechanisms.

The producers are highly adaptable and have a wide search range, guiding the population to search and forage. To obtain better fitness, scroungers follow the producers for food. At the same time, to improve the success rate of predation, some scroungers monitor the producers or forage around them. In cases where the entire population faces predators or realizes danger, the group immediately carries out collective antipredation behavior.

The core of the SSA algorithm is the producer-scrounger model, and its modeling process is as follows. Assuming that there are $N$ sparrows in a $D$-dimensional search space, the position of the $i$-th sparrow in the $D$-dimensional search space is ${X}_{i}=[{X}_{i1},{X}_{i2},\dots {X}_{iD}](i=\text{1,2}$,$\dots ,N)$, where ${X}_{id}$ represents the position of the $i$-th sparrow in the $d$-th dimension.

The producers often account for 10% to 20% of the population, and the equation for position updating is:

9 $${x}_{id}^{t+1}=\left\{\begin{array}{l}{x}_{id}^{t}\cdot exp(\frac{-i}{\alpha \cdot T}),{R}_{2}<ST\\ {x}_{id}^{t}+Q\cdot L,{R}_{2}\ge ST\end{array},\right.$$

where $t$ represents the current number of iterations, $T$ is the maximum number of iterations, $\alpha$ is a uniform random number between 0 and 1, $Q$ is a Gaussian random number, ${\varvec{L}}$ is a 1 × d matrix with all entities equal to 1, and ${R}_{2}\in [\text{0,1}]$ and $ST\in [\text{0.5,1}]$ represent the warning and safety values, respectively. In cases where ${R}_{2}<ST$, the population does not detect the presence of predators or other dangers, and the search environment is safe. Producers can extensively search to guide the population to obtain a higher degree of fitness. When ${R}_{2}\ge ST$, a sparrow detects a predator and releases the danger signal, and the population immediately performs antipredation behavior, adjusts its collective search strategy, and quickly moves closer to a safe area.

Aside from the producers, the remaining sparrows act as scroungers and update their positions according to the following formula:

10 $${x}_{id}^{t+1}=\left\{\begin{array}{l}Q\cdot exp\left(\frac{x{w}_{d}^{t}-{x}_{id}^{t}}{{i}^{2}}\right),i>\frac{n}{2}\\ x{b}_{d}^{t+1}+\frac{1}{D}\sum_{d=1}^{D}\left(\text{rand}\left\{-\text{1,1}\right\}\cdot \left|{x}_{id}^{t}-x{b}_{d}^{t+1}\right|\right),i\le \frac{n}{2}\end{array},\right.$$

where $x{w}_{d}^{t}$ represents the worst position of the given sparrow in the $d$-th dimension during the $t$-th iteration of the population, and ${x}_{id}^{t+1}$ represents the optimal position of the sparrow during the $t+1$-th iteration of the population. For $i>n/2$, the $i$-th scrounger does not obtain food and is in a state of starvation and low fitness. To obtain higher energy, it needs to fly to other places for foraging. In cases where $i\le n/2$, the $i$-th scrounger randomly finds a location near the current optimal foraging location.

#### Chaotic sparrow search optimization algorithm

The CSSA (chaotic sparrow search optimization algorithm) algorithm is improved by using Tent chaos and Gaussian mutation. Chaos, as a nonlinear phenomenon in nature, is widely used to optimize search problems due to the randomness, ergodicity, and regularity of the underlying chaotic variables^42^. This effectively maintains the diversity of the population, further helps the algorithm jump out of local optima and improves the global search performance. Through rigorous mathematical derivation, it is shown that the traversal uniformity and convergence speed of the Tent map are better than those of other forms of chaotic maps. Therefore, the CSSA algorithm uses Tent mapping to generate the chaotic sequence of the optimized algorithm. The Tent mapping expression is shown in Eq. (11). It randomly generates an initial value ${z}_{i}$ between 0 and 1 and then uses the iterative process in Eq. (11) to generate the Tent sequence.

11 $${z}_{i+1}=\left\{\begin{array}{l}2{z}_{i},0\le z\le \frac{1}{2}\\ 2(1-{z}_{i}),\frac{1}{2}<z\le 1\end{array}.\right.$$

The Gaussian variation is obtained through a Gaussian distribution, which specifically refers to replacing the original parameter value with a random number conforming to a normal distribution with mean $u$ and variance ${\sigma }^{2}$ during the mutation process.

12 $$mu\text{tation }\left(x\right)=x\left(1+N\left(\text{0,1}\right)\right),$$

where $x$ is the original parameter value, $N$(0,1) represents a normal random variable with a mean of 0 and a standard deviation of 1, and $mu\text{tation }(x)$ is the value obtained after Gaussian mutation. The normal distribution characteristics show that the key search area of Gaussian mutation is a local area near the original individual. This helps the algorithm find the global minimum point with high efficiency and high precision and improves the robustness of the algorithm^43^. In summary, the CSSA algorithm introduces the Tent chaotic search and Gaussian mutation operations. The pseudocode of this algorithm is presented below.


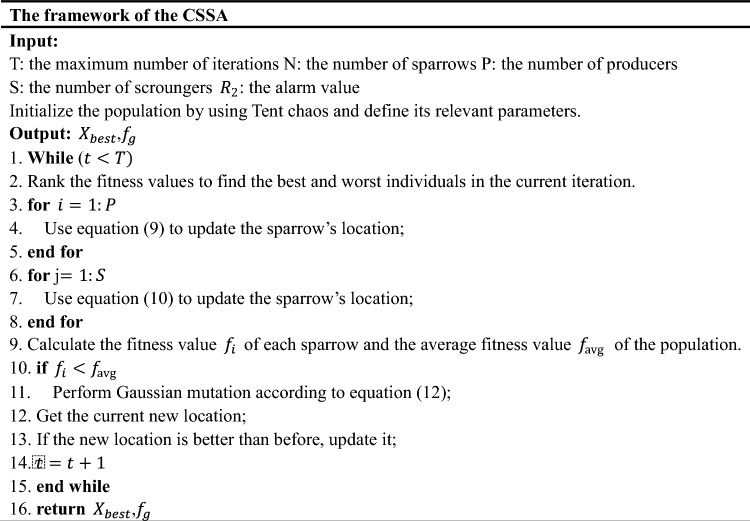


### Results and discussion

**Fig. 10:**
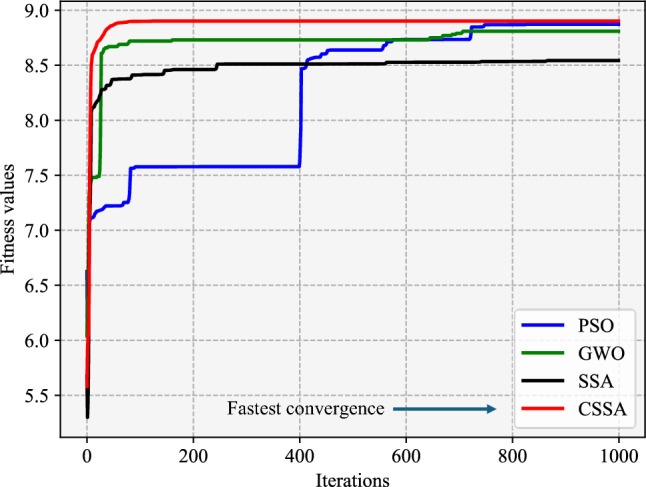
Iteration curves of the four optimization algorithms.

**Fig. 11:**
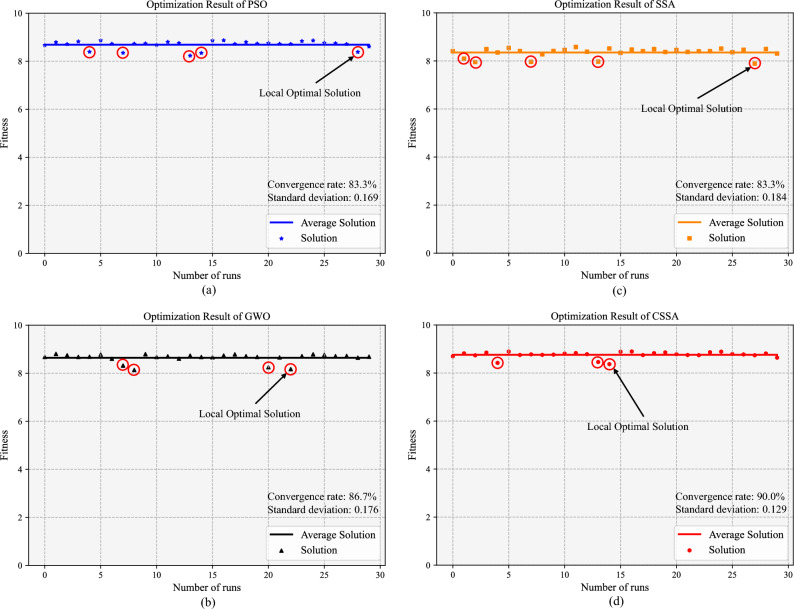
Distributions of the optimized results yielded by four algorithms.

To verify the optimized performance of the CSSA algorithm, we conduct several additional sets of optimization experiments based on the sparrow search algorithm (SSA), gray wolf optimization (GWO) algorithm, and particle swarm optimization (PSO) algorithm. To compare the convergence speeds and optimization performances of the four algorithms, we obtain the curve of the fitness value (ln(creep rupture life)) versus the number of iterations for each algorithm. As shown in Fig. 10, the CSSA has the fastest convergence rate, and its optimal solution is the best. It is also seen that the PSO and GWO algorithm evolve extremely slowly and tend to quickly reach a local optimum. Despite SSA converging faster than GWO and PSO, this premature convergence leads to it obtaining a suboptimal solution. In addition, the stability of the selected optimization algorithm is also an important criterion. Therefore, we perform 30 separate runs for each algorithm and set the number of iterations to 1000. The results in Table 3 show that the CSSA algorithm has the highest convergence rate and the lowest standard deviation. In addition, the best solution and average solution of the CSSA algorithm are the highest, and this also confirms the optimization efficiency of the CSSA. Figure 11 shows the distributions of the results for the considered optimization algorithms. The stability of the CSSA algorithm is higher than that of the other three algorithms.

**Table 3:** Statistical results obtained by four different optimization algorithms.

Optimization algorithm	Convergence rate	The best solution	The average solution	Standard deviation
PSO	25/30	8.8733	8.6878	0.16928
GWO	26/30	8.8121	8.6429	0.17557
SSA	25/30	8.5424	8.3536	0.18396
CSSA	27/30	8.9014	8.7559	0.12881

Table 4 shows the best combination of these 27 features obtained by the CSSA algorithm. Initially, we observe that the sum of the chemical element contents (i.e., the numerical values of the first fourteen features) comprising Ni-based single crystal superalloy amounts to 100%, fulfilling the equality constraint specified in model (8). Aside from Ni, Ta has the largest proportion of approximately 11.872%. This is also consistent with our conclusion in “Explanatory methods for interpreting creep rupture life” section that the creep life is positively correlated with the Ta content. Therefore, in industrial production processes, increasing the Ta content is essential for improving the creep rupture life. Moreover, the processing parameters are equally important to the creep rupture life, such as the second-stage aging treatment time (2satt) and the first-stage aging treatment time (1satt). This further suggests that adjustment of 1satt and 2satt enhances the creep resistance of the tested alloy. It should be noted that when all the features are fully considered, a satisfactory creep rupture life can be achieved.

**Table 4:** One of the optimization results of the CSSA.

Ni (%)	Re (%)	Co (%)	Al (%)	Ti (%)	W (%)	Mo (%)	Cr (%)	Ta (%)
60.199	2.163	0.101	4.150	3.227	10.485	0.674	4.399	11.872
C (%)	B (%)	Y (%)	Nb (%)	Hf (%)	Stt (h)	1satt (h)	2satt (h)	StT (°C)
0.025	0.048	0.025	2.444	0.188	3.139	4.138	22.524	1299.178
1satT (°C)	2satT (°C)	T (°C)	*S* (MPa)	$\Gamma$ (${\text{mJ}}/{\text{m}}^{2}$)	${D}_{L}$ (${\text{m}}^{2}/{\text{s}}$)	$G$ (GPa)	$L$ (nm)	${\text{X}}^{{\gamma \prime}}$ (–)
1104.011	885.346	893.655	119.791	41.332	0.001	67.710	0.343	0.596

## Conclusions

In the field of developing new materials and improving material compositions, the data-driven approach offers irreplaceable advantages. Many of the previous studies on creep rupture life focused on performing large numbers of experiments, but this is not cost-effective and requires considerable time. With the accumulation of industrial big data, machine learning can provide a simpler and more efficient solution for creep life prediction and optimization. In this paper, we establish 10 different machine learning models to predict creep fracture life and finally select XGBoost by comparing the accuracy rates of the tested models. To gain insights into black-box ML models, we then propose using the SHAP model to explain the working mechanism of creep rupture life. The process of interpretation is divided into two steps: (1) visualizing the positive and negative feature contributions of each feature using the corresponding SHAP values and (2) ranking the importance levels of features based on global interpretation methods. Ultimately, we obtain an optimal combination of 27 features with the chaotic sparrow search optimization algorithm. In summary, the proposed method can provide theoretical guidance for improving creep life. In future works, we will extend the approach in this paper by incorporating other parameters, such as activation energy and lattice misfit.

## Data Availability

The datasets used during the current study are available from the corresponding author on reasonable request.

## References

[1] Zhang, Y. & Xu, X. Lattice misfit predictions via the Gaussian process regression for Ni-based single crystal superalloys. *Met. Mater. Int.*10.1007/s12540-020-00883-7 (2020). 10.1007/s12540-020-00883-7

[2] Choi, B. G. *et al.* Effect of Ti content on creep properties of Ni-base single crystal superalloys. *Met. Mater. Int.* **23**, 877–883. 10.1007/s12540-017-7089-7 (2017). 10.1007/s12540-017-7089-7

[3] Rajan, K. Materials informatics. *Mater. Today* **8**, 38–45. 10.1016/S1369-7021(05)71123-8 (2005). 10.1016/S1369-7021(05)71123-8

[4] Reed, R. C., Tao, T. & Warnken, N. Alloys-by-design: Application to nickel-based single crystal superalloys. *Acta Mater.* **57**, 5898–5913. 10.1016/j.actamat.2009.08.018 (2009). 10.1016/j.actamat.2009.08.018

[5] Terada, Y. & Sato, T. Assessment of creep rupture life of heat resistant Mg–Al–Ca alloys. *J. Alloys Compd.* **504**, 261–264. 10.1016/j.jallcom.2010.05.108 (2010). 10.1016/j.jallcom.2010.05.108

[6] Li, S., Wang, B., Shi, D., Yang, X. & Qi, H. A physically based model for correlating the microstructural degradation and residual creep lifetime of a polycrystalline Ni-based superalloy. *J. Alloys Compd.* **783**, 565–573. 10.1016/j.jallcom.2018.11.417 (2019). 10.1016/j.jallcom.2018.11.417

[7] Dang, Y. Y. *et al.* Predicting long-term creep-rupture property of Inconel 740 and 740H. *Mater. High Temp.* **33**(1), 1–5. 10.1179/1878641315Y.0000000010 (2016). 10.1179/1878641315Y.0000000010

[8] Bolton, J. Reliable analysis and extrapolation of creep rupture data. *Int. J. Press. Vessels Pip.* **157**, 1–19. 10.1016/j.ijpvp.2017.08.001 (2017). 10.1016/j.ijpvp.2017.08.001

[9] Hong, D., Kwon, S. & Yim, C. Exploration of machine learning to predict hot ductility of cast steel from chemical composition and thermal conditions. *Met. Mater. Int.*10.1007/s12540-020-00713-w (2020). 10.1007/s12540-020-00713-w

[10] Thankachan, T., Soorya Prakash, K., Kavimani, V. & Silambarasan, S. R. Machine learning and statistical approach to predict and analyze wear rates in copper surface composites. *Metals Mater. Int.* **27**, 220. 10.1007/s12540-020-00809-3 (2020). 10.1007/s12540-020-00809-3

[11] Lin, Y. C., Yang, H., Chen, D.-D. & He, D.-G. Stacked auto-encoder network to predict tensile deformation behavior of a typical nickel-based superalloy considering Portevin–Le Chatelier effects. *Met. Mater. Int.*10.1007/s12540-019-00435-8 (2019). 10.1007/s12540-019-00435-8

[12] Liu, Y., Zhao, T., Ju, W. & Shi, S. Materials discovery and design using machine learning. *J. Materiom.* **3**, 159–177. 10.1016/j.jmat.2017.08.002 (2017). 10.1016/j.jmat.2017.08.002

[13] Jain, D. K., Jain, R., Lan, X., Upadhyay, Y. & Thareja, A. Driver distraction detection using capsule network. *Neural Comput. Appl.* **1**, 1–14. 10.1007/s00521-020-05390-9 (2020). 10.1007/s00521-020-05390-9

[14] Jain, D. K., Lan, X. & Manikandan, R. Fusion of iris and sclera using phase intensive rubbersheet mutual exclusion for periocular recognition. *Image Vis. Comput.* **103**, 104024. 10.1016/j.imavis.2020.104024 (2020). 10.1016/j.imavis.2020.104024

[15] Jain, D., Jain, R., Upadhyay, Y., Kathuria, A. & Lan, X. Deep refinement: Capsule network with attention mechanism-based system for text classification. *Neural Comput. Appl.* **32**, 1. 10.1007/s00521-019-04620-z (2020). 10.1007/s00521-019-04620-z

[16] Jain, D., Kumar, A. & Garg, G. Sarcasm detection in mash-up language using soft-attention based bi-directional LSTM and feature-rich CNN. *Appl. Soft Comput.* **91**, 106198. 10.1016/j.asoc.2020.106198 (2020). 10.1016/j.asoc.2020.106198

[17] Venkatesh, V. & Rack, H. J. A neural network approach to elevated temperature creep–fatigue life prediction. *Int. J. Fatigue* **21**, 225–234. 10.1016/S0142-1123(98)00071-1 (1999). 10.1016/S0142-1123(98)00071-1

[18] Yoo, Y. S., Jo, C. Y. & Jones, C. N. Compositional prediction of creep rupture life of single crystal Ni base superalloy by Bayesian neural network. *Mater. Sci. Eng. A* **336**, 22–29. 10.1016/S0921-5093(01)01965-7 (2002). 10.1016/S0921-5093(01)01965-7

[19] Liu, Y. *et al.* Predicting creep rupture life of Ni-based single crystal superalloys using divide-and-conquer approach based machine learning. *Acta Mater.* **195**, 454–467. 10.1016/j.actamat.2020.05.001 (2020). 10.1016/j.actamat.2020.05.001

[20] Conduit, B. D., Jones, N. G., Stone, H. J. & Conduit, G. J. Design of a nickel-base superalloy using a neural network. *Mater. Des.* **131**, 358–365. 10.1016/j.matdes.2017.06.007 (2017). 10.1016/j.matdes.2017.06.007

[21] Yamazaki, M., Yamagata, T. & Harada, H. *Nickel-Base Single Crystal Superalloy and Process for Production Thereof, US Patent, 4707192* 1–12 (1987).

[22] Milligan, G. W. & Cooper, M. C. A study of standardization of variables in cluster analysis. *J. Classif.* **5**, 181–204. 10.1007/BF01897163 (1988). 10.1007/BF01897163

[23] Chen, T. & Guestrin, C. Xgboost: A scalable tree boosting system. In *Proc. 22nd ACM SIGKDD International Conference on Knowledge Discovery and Data Mining* 785–794. 10.1145/2939672.2939785 (ACM, 2016).

[24] Rao, H. *et al.* Feature selection based on artificial bee colony and gradient boosting decision tree. *Appl. Soft Comput.* **74**, 634–642. 10.1016/j.asoc.2018.10.036 (2019). 10.1016/j.asoc.2018.10.036

[25] Sutskever, I., Hinton, G. E. & Krizhevsky, A. Imagenet classification with deep convolutional neural networks. *Adv. Neural. Inf. Process. Syst.*10.1145/3065386 (2012). 10.1145/3065386

[26] Weber, G., Pinz, M. & Ghosh, S. Machine learning-aided parametrically homogenized crystal plasticity model (PHCPM) for single crystal Ni-based superalloys. *JOM* **72**, 4404–4419. 10.1007/s11837-020-04344-9 (2020). 10.1007/s11837-020-04344-9

[27] Rutkowski, L., Jaworski, M., Pietruczuk, L. & Duda, P. The CART decision tree for mining data streams. *Inf. Sci.* **266**, 1–15. 10.1016/j.ins.2013.12.060 (2014). 10.1016/j.ins.2013.12.060

[28] Abe, S. Variants of support vector machines. In *Support Vector Machines for Pattern Classification* (ed. Abe, S.) 163–226 (Springer, 2010).

[29] Liu, C.-L., Fink, G. A., Govindaraju, V. & Jin, L. Special issue on deep learning for document analysis and recognition. *Int. J. Doc. Anal. Recogn.* **21**(3), 159–160. 10.1007/s10032-018-0310-5 (2018). 10.1007/s10032-018-0310-5

[30] Breiman, L. Random Forests. *Mach. Learn.* **45**, 5–32. 10.1023/A:1010933404324 (2001). 10.1023/A:1010933404324

[31] Sun, X., Liu, M. & Sima, Z. A novel cryptocurrency price trend forecasting model based on LightGBM. *Financ. Res. Lett.* **32**, 101084. 10.1016/j.frl.2018.12.032 (2020). 10.1016/j.frl.2018.12.032

[32] Bisong, E. & Bisong, E. *Introduction to Scikit-Learn. Building Machine Learning and Deep Learning Models on Google Cloud Platform: A Comprehensive Guide for Beginners* 215–229 (2019).

[33] Akiba, T. et al. Optuna: A next-generation hyperparameter optimization framework. In *Proc. 25th ACM SIGKDD International Conference on Knowledge Discovery & Data Mining* 2623–2631 (2019).

[34] Lisboa, P. J. G. Interpretability in machine learning—Principles and practice. In *Fuzzy Logic and Applications* (eds Masulli, F. *et al.*) 15–21 (Springer, 2013).

[35] Lundberg, S. M. & Lee, S.-I. A unified approach to interpreting model predictions. In *Proc. 31st International Conference on Neural Information Processing Systems* 4768–4777. http://arxiv.org/abs/1705.07874 (Curran Associates Inc., 2017).

[36] de Sousa, I. P. *et al.* Local interpretable model-agnostic explanations for classification of lymph node metastases. *Sensors* **19**, 969. 10.3390/s19132969 (2019). 31284419 10.3390/s19132969 PMC6651753

[37] Nowak, A. S. & Radzik, T. The Shapley value for n-Person games in generalized characteristic function form. *Games Econom. Behav.* **6**, 150–161. 10.1006/game.1994.1008 (1994). 10.1006/game.1994.1008

[38] Xue, J. & Shen, B. A novel swarm intelligence optimization approach: Sparrow search algorithm. *Syst. Sci. Control Eng.* **8**, 22–34. 10.1080/21642583.2019.1708830 (2020). 10.1080/21642583.2019.1708830

[39] Sibalija, T. V. Particle swarm optimisation in designing parameters of manufacturing processes: A review (2008–2018). *Appl. Soft Comput.* **84**, 105743. 10.1016/j.asoc.2019.105743 (2019). 10.1016/j.asoc.2019.105743

[40] Dorigo, M. & Blum, C. Ant colony optimization theory: A survey. *Theoret. Comput. Sci.* **344**, 243–278. 10.1016/j.tcs.2005.05.020 (2005). 10.1016/j.tcs.2005.05.020

[41] Mirjalili, S., Mirjalili, S. M. & Lewis, A. Grey wolf optimizer. *Adv. Eng. Softw.* **69**, 46–61. 10.1016/j.advengsoft.2013.12.007 (2014). 10.1016/j.advengsoft.2013.12.007

[42] Liu, L., Sun, S. Z., Yu, H., Yue, X. & Zhang, D. A modified fuzzy C-means (FCM) clustering algorithm and its application on carbonate fluid identification. *J. Appl. Geophys.* **129**, 28–35. 10.1016/j.jappgeo.2016.03.027 (2016). 10.1016/j.jappgeo.2016.03.027

[43] Rudolph, G. Local convergence rates of simple evolutionary algorithms with Cauchy mutations. *IEEE Trans. Evol. Comput.* **1**, 249–258. 10.1109/4235.687885 (1997). 10.1109/4235.687885

